# Supplementary material for: Modulation of GCN2/eIF2α/ATF4 Pathway in the Liver and Induction of FGF21 in Young Goats Fed a Protein- and/or Phosphorus-Reduced Diet
Source: Int J Mol Sci. 2023 Apr 12;24(8):7153. doi: 10.3390/ijms24087153 (PMC10138370; doi:10.3390/ijms24087153)
Supplement: Supplementary file 1 [file ijms-24-07153-s001.zip › ijms-2298230-supplementary.pdf]

Table S1. Correlations between circulating plasma EAA and NEAA and plasma urea, P_i, serum FGF21, *GCN2*, *ATF4*, *FGF21* mRNA expression, and GCN2 protein expression in young goats fed an N- and/or P-reduced diet.

Variable	r/*p*	Plasma Urea	Plasma P_i	GCN2 protein	*GCN2* mRNA	*ATF4* mRNA	*FGF21* mRNA	Serum FGF21
Threonine	r	0.494	-0.377	-0.550	-0.448	-0.519	-0.652	-0.648
	p	0.008	0.048	0.002	0.017	0.005	0.0002	0.0002
Valine	r	0.564	-0.247	-0.511	-0.349	-0.484	-0.517	-0.651
	p	0.002	0.205	0.005	0.069	0.009	0.005	0.0002
Methionine	r	0.066	0.121	0.016	0.236	0.004	0.154	-0.025
	p	0.739	0.542	0.936	0.226	0.986	0.435	0.899
Isoleucine	r	0.370	-0.262	-0.3828	-0.354	-0.408	-0.409	-0.492
	p	0.053	0.177	0.044	0.065	0.031	0.031	0.008
Leucine	r	0.641	-0.421	-0.505	-0.360	-0.504	-0.479	-0.516
	p	0.0002	0.026	0.006	0.060	0.006	0.010	0.005
Phenylalanine	r	-0.645	-0.055	0.107	0.244	0.303	0.346	0.331
	p	0.0002	0.782	0.590	0.211	0.117	0.072	0.086
Lysine	r	0.638	-0.124	-0.527	-0.270	-0.5101	-0.472	-0.382
	p	0.0003	0.530	0.004	0.165	0.006	0.011	0.045
Histidine	r	-0.326	-0.107	-0.181	0.082	0.054	-0.156	-0.163
	p	0.091	0.587	0.356	0.678	0.784	0.429	0.408
Arginine	r	0.292	-0.243	-0.436	-0.300	-0.340	-0.548	-0.335
	p	0.132	0.212	0.020	0.121	0.077	0.003	0.082
Serine	r	-0.315	0.190	0.183	0.453	0.302	0.385	0.219
	p	0.102	0.360	0.351	0.016	0.119	0.043	0.263
Aspartic acid	r	-0.036	0.130	-0.145	0.114	-0.030	-0.056	-0.326
	p	0.854	0.511	0.463	0.565	0.880	0.776	0.090
Glutamine	r	-0.447	0.264	0.505	0.466	0.483	0.626	0.268
	p	0.017	0.175	0.006	0.012	0.009	0.0004	0.167
Glutamic acid	r	0.275	0.162	-0.257	0.129	-0.056	-0.113	-0.468
	p	0.157	0.412	0.186	0.512	0.778	0.567	0.012
Glycine	r	-0.649	0.176	0.495	0.505	0.564	0.584	0.285
	p	0.0002	0.370	0.007	0.006	0.002	0.001	0.141
Alanine	r	-0.808	0.304	0.518	0.605	0.621	0.675	0.456
	p	<0.0001	0.115	0.005	0.001	0.0004	<0.0001	0.015
Tyrosine	r	-0.777	0.108	0.408	0.374	0.457	0.534	0.402
	p	<0.0001	0.585	0.031	0.050	0.015	0.003	0.034
Proline	r	-0.666	-0.092	0.275	-0.020	0.0956	0.189	0.216
	p	0.0001	0.643	0.157	0.918	0.628	0.336	0.269

EAA (essential amino acids), NEAA (non-essential amino acids), P_i (inorganic phosphate), FGF21 (fibroblast growth factor 21), GCN2 (general control nonderepressible 2), ATF4 (activating transcription factor 4). $p < 0.05$. n = 28 animals.
